# Head‐and‐neck multichannel B1 ^+^ mapping and RF shimming of the carotid arteries using a 7T parallel‐transmit head coil

**DOI:** 10.1002/mrm.29845

**Published:** 2023-10-05

**Authors:** Matthijs H. S. de Buck, James L. Kent, Peter Jezzard, Aaron T. Hess

**Affiliations:** ^1^ FMRIB Division, Nuffield Department of Clinical Neurosciences, Wellcome Centre for Integrative Neuroimaging University of Oxford Oxford UK

**Keywords:** B_1_^+^ mapping, MRI, neurovascular, parallel transmission, RF shimming

## Abstract

**Purpose:**

Neurovascular MRI suffers from a rapid drop in B_1_^+^ into the neck when using transmit head coils at 7 T. One solution to improving B_1_^+^ magnitude in the major feeding arteries in the neck is to use custom RF shims on parallel‐transmit head coils. However, calculating such shims requires robust multichannel B_1_^+^ maps in both the head and the neck, which is challenging due to low RF penetration into the neck, limited dynamic range of multichannel B_1_^+^ mapping techniques, and B_0_ sensitivity. We therefore sought a robust, large‐dynamic‐range, parallel‐transmit field mapping protocol and tested whether RF shimming can improve carotid artery B_1_^+^ magnitude in practice.

**Methods:**

A pipeline is presented that combines B_1_^+^ mapping data acquired using circularly polarized (CP) and CP2‐mode RF shims at multiple voltages. The pipeline was evaluated by comparing the predicted and measured B_1_^+^ for multiple random transmit shims, and by assessing the ability of RF shimming to increase B_1_^+^ in the carotid arteries.

**Results:**

The proposed method achieved good agreement between predicted and measured B_1_^+^ in both the head and the neck. The B_1_^+^ magnitude in the carotid arteries can be increased by 43% using tailored RF shims or by 37% using universal RF shims, while also improving the RF homogeneity compared with CP mode.

**Conclusion:**

B_1_^+^ in the neck can be increased using RF shims calculated from multichannel B_1_^+^ maps in both the head and the neck. This can be achieved using universal phase‐only RF shims, facilitating easy implementation in existing sequences.

## 1 INTRODUCTION

Ultrahigh‐field MRI offers increased SNR and longer T_1_ relaxation time for both tissue and blood. At 7 T, these properties have the potential to improve the contrast, resolution, and imaging time of intracranial neurovascular modalities such as cerebral angiography and perfusion, as well as the visualization of vessel wall pathology. However, increasing B_0_ also introduces limitations due to increased specific absorption rate (SAR) and reduced homogeneity and spatial extent of the transmit magnetic field (B_1_^+^).^1^

When using typical transmit head coils at 7 T, intracranial neurovascular imaging methods such as arterial spin labeling (ASL)^2^, ^3^, ^4^, ^5^, ^6^ and intracranial vessel wall imaging^7^, ^8^ suffer from the rapid drop of B_1_^+^ into the neck. For ASL, this drop in B_1_^+^ limits the ability to effectively invert the inflowing blood in upstream labeling planes, such as in the carotid arteries in the neck.^3^, ^4^, ^5^, ^6^ For vessel wall imaging, it reduces the ability to suppress the signal in upstream arterial blood, which is required to provide sufficient black‐blood contrast between the vessel wall and the inflowing arterial blood. Although higher nominal flip angles can be applied to increase the inversion or saturation efficiency of arterial blood in low‐B_1_^+^ areas, this is in practice constrained by a quadratic increase in SAR and by adverse effects on the magnetization of stationary spins within higher B_1_^+^ imaging regions. Dielectric pads^9^, ^10^ can be positioned near the neck to increase both the transmit and receive sensitivity,^3^, ^11^ which has been found to increase the B_1_^+^ efficiency in a slice just below the carotid siphon by 57%.^3^ However, the use of dielectric pads increases experimental complexity and does not provide the ability to change the B_1_^+^ field over time, such as between different acquisitions or between signal preparation and readout modules within a single acquisition.

Improved control over B_1_^+^ in the neck can also be achieved using parallel transmission (pTx)^12^ coils, which consist of multiple separate transmit channels. pTx provides improved control over the B_1_^+^ field by manipulating the amplitude and/or phase of each of the individual transmit channels, known as RF shimming. This can be used to achieve improved spatial homogeneity of the B_1_^+^ field,^13^ to reduce the SAR, to achieve spatial^14^, ^15^ and spectral^16^ selectivity, or to increase the B_1_^+^ magnitude within a particular region of interest. Therefore, the use of pTx coils for neurovascular imaging could be used to improve the B_1_^+^ in the feeding arteries in the neck, thereby allowing improved inversion or saturation of inflowing arterial blood.

Most conventional head pTx coils are designed for imaging the brain and lack transmit penetration into the neck. To improve the B_1_^+^ coverage in the brainstem, the cerebellum, and the carotid arteries, previous work proposed custom pTx coil designs that consist of transmit elements surrounding both the brain and the neck (using fixed^17^ or geometrically adjustable^18^ transmit arrays). However, the use of such coil designs adds experimental complexity and expense relative to the use of conventional pTx coil designs. Therefore, this work focuses on the potential of improving B_1_^+^ in the neck using conventional head pTx coils.

Before it is possible to calculate and optimize the achieved B_1_^+^ field using pTx methods, the transmit field of all individual pTx channels must be characterized. Such multichannel B_1_^+^ mapping^19^ aims to measure the transmitted magnitude and relative phase of each transmit channel. Acquiring these data in both the head and the neck using pTx head coils can be challenging due to a combination of low RF penetration into the neck and the inherently limited dynamic range^19^ of B_1_^+^ mapping techniques. Using B_1_^+^ mapping techniques with typical transmit voltages provides accurate data in the brain but does not provide useful information in the low‐B_1_^+^ areas in the neck. Conversely, using high transmit voltages can achieve improved B_1_^+^ coverage in the neck, but is inaccurate for high‐B_1_^+^ regions in the head.

This paper proposes and validates an approach that combines B_1_^+^ mapping data acquired at 7 T using two complementary RF shims (to ensure adequate overall coverage) and at multiple transmit voltages, to allow robust reconstruction of multichannel B1^+^ maps for both the head and the neck. Subsequently, data acquired using the proposed method are used to investigate how RF shimming can be used to improve the B_1_^+^ magnitude in the major feeding arteries in the neck.

Parts of this work have previously been presented at the annual meeting of the International Society for Magnetic Resonance in Medicine.^20^, ^21^

## 2 THEORY

Multichannel absolute B_1_^+^ maps can be obtained through direct measurement of (linear combinations of) the absolute B_1_^+^ fields of the individual transmit elements. However, this is time‐consuming and requires high sensitivity for the individual acquisitions. Alternatively, a combined absolute B_1_^+^ map (${B1}_{\mathrm{abs}}^{+}$) can be acquired for all *n* transmit elements, together with a full set of relative B_1_^+^ maps (${B1}_{\mathrm{rel}}^{+}$), where the relative contribution of each transmit element is estimated. Using this, the absolute B_1_^+^ field for the ith transmit element can be calculated based on its relative contribution to the combined absolute B_1_^+^ field, as follows:

(1) $${B1}_{i}^{+}=\frac{{B1}_{\mathrm{rel},i}^{+}}{\sum_{j=1}^{n}{B1}_{\mathrm{rel},j}^{+}}{B1}_{\mathrm{abs}}^{+},$$

where the calculated ${B1}_{i}^{+}$ values correspond to the shim configuration used for ${B1}_{\mathrm{abs}}^{+}$. Relative B_1_^+^ maps are acquired based on the signal ratios of spoiled gradient echo (SPGR) images acquired from the individual transmit channels. In the low‐flip‐angle regime, SPGR signal levels are proportional to transmit field magnitudes. However, with large spatial variation of the transmit field strength (such as between the head and the neck at 7 T), this low‐flip‐angle assumption does not apply over the full FOV. Therefore, Padormo et al.^22^ proposed combining SPGR data from different RF transmission voltages. For the jth transmission voltage, the measured ratio between the steady‐state SPGR signal $S_{j}$ and the applied transmission voltage $d_{j}$ can be expressed using the low‐flip‐angle approximations as

(2) $$\frac{S_{j}}{d_{j}}=I_{\text{prop}}+\frac{\varepsilon }{d_{j}}+\frac{n_{j}}{d_{j}},$$

where $I_{\text{prop}}\propto {B1}_{\mathrm{rel}}^{+}$ is the desired B_1_^+^‐proportional image intensity; ε denotes the Gaussian noise contribution; and $n_{j}$ (which is always negative) corresponds to systematic errors due to the low‐flip‐angle approximation in the presence of saturation effects. Using this equation, the B_1_^+^‐proportional image component of each individual voxel is calculated for each transmit channel using maximum likelihood estimation^23^ across the data sets acquired at different transmission voltages.

**FIGURE 1:**
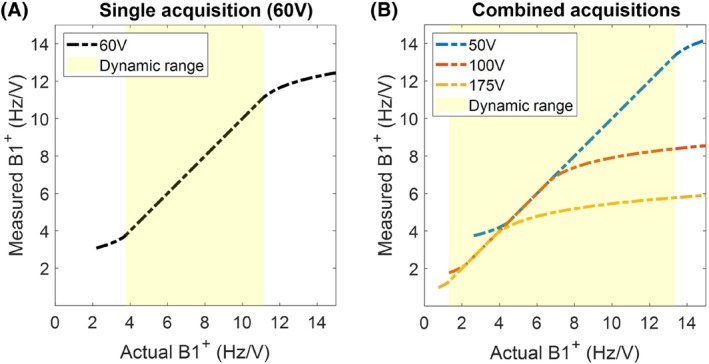
Schematic representation of the measured absolute B_1_^+^ values for acquisitions with different transmit voltages. Each single acquisition has a linear response within its dynamic range. Higher B_1_^+^ magnitudes result in underestimation of the B_1_^+^; lower values result in either overestimated or noise‐dominated B_1_^+^ values. Subplots show a single acquisition using a transmit voltage of 60 V (A) and the larger combined dynamic range when using three separate acquisitions at 50, 100, and 175 V (B).

We propose using a similar approach to increase the dynamic range of absolute B_1_^+^ measurements by combining acquisitions using different transmit voltages (50, 100, and 175 V per channel). The B_1_^+^ maps are acquired using a 3D B_1_^+^ mapping method termed Sandwiched satTFL,^24^ which has a reported dynamic range of 40° to 120° (at a per‐channel voltage of 60 V). We increase this dynamic range by acquiring data at different transmit voltages with overlap in the dynamic range of the maps (Figure 1). We propose a series of consistency criteria to identify which voxels in each map are within the identified dynamic range. The individual reconstructions are first expressed in voltage‐independent units of Hz/V. Then, on a voxel‐by‐voxel basis, measured values are excluded from the final (combined) B_1_^+^ map:

1. If the satTFL reference image ($S_{0}$) signal is smaller than the noise SD; or
2. If the measured value at a given transmit voltage is outside the dynamic range (< 4.44 Hz/V at 50 V per channel, < 2.22 Hz/V or > 6.67 Hz/V at 100 V per channel, or > 3.81 Hz/V at 175 V per channel); or
3. If more than one value remains for a given voxel, the most appropriate value(s) is/are retained as follows:
  - Some (< 1/1000) voxels gave values within the expected dynamic ranges at all three voltages, despite the valid dynamic ranges not overlapping. This generally occurred at air/tissue interfaces, and these voxels were therefore masked to prevent unreliable values.
  - If either measurements at both 50 V and 100 V or both 100 V and 175 V are valid for a given voxel, this is the result of respective overlapping dynamic ranges. If the average value is in the higher half of the overlapping range, only the measurement at the higher transmit voltage is used to maintain higher SNR. Otherwise, both values are included in the calculation of the combined map.

If only one transmit voltage value remained for a given voxel after applying all three exclusion criteria, that value was used for the combined B_1_^+^ map. If multiple transmit voltages remained (due to being within the lower half of the overlapping dynamic range), the average value of the B_1_^+^ measurements was used.

Inaccuracies in absolute B_1_^+^ data reconstructed from different transmit voltages can remain in locations with low B_1_^+^ magnitudes due to destructive interference of the individual transmit channels. To provide increased coverage in areas with low B_1_^+^ for any given shim, methods such as B_1_^+^ time‐interleaved acquisition of modes (B1TIAMO^25^) can be used to combine B_1_^+^ maps acquired using different RF shims. B1TIAMO combines acquisitions using different RF shims as a weighted average of the B_1_^+^ based on the signal levels of the respective reference images to provide a single combined reconstruction with more consistent accuracy.

Finally, additional B_0_ correction can be required when RF pulses used in the B_1_^+^ mapping sequence have a frequency dependence. For example, when using a 500‐μs rectangular pulse for presaturation (as used in this paper), the frequency response is a sinc function with zero‐crossings at ±2 kHz. However, if the frequency dependence of such an RF pulse is known, its effects can be corrected (at the cost of an SNR penalty proportional to the frequency dependence) by scaling the voxel‐wise B_1_^+^ estimates based on a separately acquired B_0_ map. Note that, since this work, the sandwiched satTFL implementation has been proposed to use a B_0_‐insensitive hyperbolic secant pulse.^24^

## 3 METHODS

### 3.1 Wide dynamic range multichannel B_1_ ^+^ mapping

**FIGURE 2:**
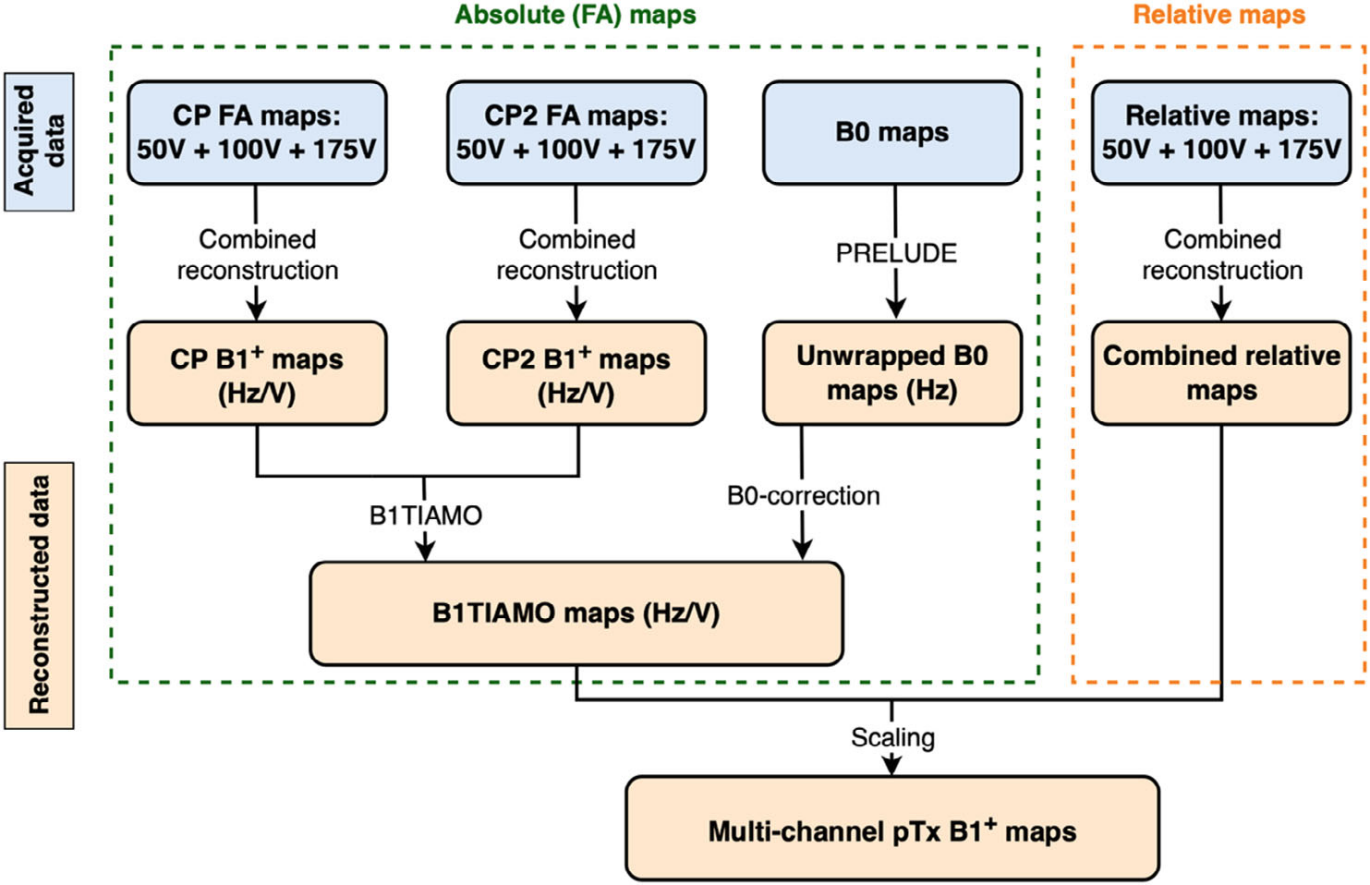
Schematic of the proposed B_1_^+^ map acquisition and processing pipeline. In total, 10 different data sets are acquired (blue: six flip angle [FA] maps, one B_0_ map, and three sets of relative maps) to reconstruct a single set of multichannel B_1_^+^ maps. B1TIAMO, B_1_^+^ time‐interleaved acquisition of modes; CP, circularly polarized; pTx, parallel transmission.

To obtain robust multichannel B_1_^+^ maps, a three‐step process was used, as summarized in Figure 2.

For the first step, sandwiched satTFL absolute B_1_^+^ maps were acquired at three different transmit voltages (50, 100, and 175 V per channel) to achieve the required large dynamic range. Absolute B_1_^+^ maps at all three voltages were acquired twice, using two complementary RF shim configurations (circular polarization or CP mode [defined by the coil manufacturer] and CP2‐mode [defined as a 45° phase increment per channel on top of CP mode]) and combined using the B1TIAMO^25^ postprocessing approach. This approach calculates a weighted average of the B_1_^+^ maps acquired using the two RF shims. For this, a B1TIAMO weighting factor m, as used in Eq. (4) of Brunheim et al.,^25^ of 3 was used based on preliminary results. A B_0_ map was also acquired to correct for static field‐inhomogeneity effects arising from the 500‐μs rectangular preparation RF pulse used for presaturated TurboFLASH B_1_^+^ mapping. All acquired absolute B_1_^+^ maps and the B_0_ map were combined to form a single absolute B_1_^+^ map in Hz/V. Second, relative B_1_^+^ maps were acquired at multiple voltages (50, 100, and 175 V per channel) and reconstructed using the previously described large dynamic range relative B_1_^+^ mapping approach.^22^ The final step combined the absolute maps with the relative maps to form complex multichannel B_1_^+^ field maps in both the head and neck (Eq. [1]; final step in Figure 2).

Scan parameters for each individual 3D sandwiched satTFL acquisition include TR = 1 s, time domain = 0 s,^20^ TE = 1.78 ms, nominal flip angle = 9°, nominal preparation flip angle = 90° (using a 500‐μs rectangular pulse), bandwidth = 489 Hz/px, and scan time = 36 s.

B_0_ maps were acquired using a 3D gradient‐recalled echo acquisition with TR = 4.9 ms, TE_1_/TE_2_ = 1.02/3.06 ms, nominal flip angle = 7°, bandwidth = 538 Hz/px, and scan time = 1:39 min. PRELUDE^26^ was used to unwrap the phase maps used for B_0_ measurement.^23^

Relative transmit maps were acquired using low‐flip‐angle SPGR acquisitions (TR = 2.90 ms, TE = 1.02 ms, nominal flip angle = 7°, bandwidth = 500 Hz/px, and scan time = 30 s per single voltage acquisition). Transmit channels were excited in an interleaved fashion (with the channels ordered as 1‐6‐2‐5‐3‐8‐4‐7) R, to minimize magnetization history effects.

### 3.2 In vivo experiments

Data were acquired in 10 healthy volunteers (23–56 years old; 8 male/2 female). All acquisitions (B_1_^+^, B_0_, and structural data) were performed in 3D using the same FOV (225 × 225 × 300 mm) in the same absolute coordinates relative to the coil. MPRAGE structural data were acquired at 1.2‐mm isotropic resolution for anatomical reference. Other MPRAGE scan parameters include TR = 2200 ms, TE = 2.77 ms, TI = 1050 ms, flip angle = 7°, bandwidth = 238 Hz/px, and scan time = 3:57 min. MPRAGE data were reconstructed as the root sum of squares of separate data sets acquired in CP mode and CP2 mode, to improve the coverage into the neck of the structural information. For B_1_^+^ and B_0_ field maps, a lower resolution of 7.5 × 5.6 × 6.2 mm per voxel was used.

Data were acquired on a Siemens (Erlangen, Germany) Magnetom 7T scanner using a Nova Medical (Wilmington, MA) 8Tx/32Rx head coil under an institutional ethics agreement. To ensure consistency in the acquired B_0_ data, the tune‐up B_0_ shim was used for all acquisitions. Data reconstruction and shim calculation were performed using *MATLAB* (The MathWorks, Natick, MA, USA) on a system using an Intel (Santa Clara, CA, USA) Xeon CPU E5‐2680 (v4) running at 2.40 GHz with 14 cores and 28 logical processors.

Using the approach outlined in Figure 2, large dynamic range B_1_^+^ field maps were measured and reconstructed for all 10 volunteers. For 4 subjects (Subjects 1, 2, 9, and 10), additional absolute B_1_^+^ maps were measured for validation purposes using two arbitrary RF shims (again acquired at reference voltages of 50, 100, and 175 V per channel to facilitate validation with full spatial coverage).

### 3.3 Carotid artery RF shimming

To assess the theoretical upper limit for the boost in B_1_^+^ in the neck that can be achieved using pTx RF shims, the total (theoretically) available B_1_^+^ was evaluated in vivo on a voxel‐by‐voxel basis by summing the B_1_^+^ magnitudes across the transmit channels.

For shim calculations and evaluation, hand‐drawn vessel masks, comprising the internal carotid arteries (ICAs) and the circle of Willis, were drawn for each subject from the MPRAGE images. These regions of interest were down‐sampled to the resolution of the B_1_^+^ and B_0_ data and used as masks for the RF shim calculations. Where needed, the regions of interest were reduced to the areas corresponding to the carotid arteries.

Both phase‐and‐magnitude and phase‐only RF shim combinations were calculated to assess any potential benefit of the extra degrees of freedom. Shims were calculated using cost functions that aim to maximize either B_1_^+^ magnitude, B_1_^+^ homogeneity, or a combination of both. The cost function $\min\left\{<{\left(1/B1^{+}\right)}^{2}>\right\}$ was used to maximize the magnitude, where a quadratic term is used to ensure simultaneous minimization of the required energy (as a surrogate for global SAR) to achieve a certain effective flip angle. The coefficient of variation (CoV) was used to maximize the B_1_^+^ homogeneity: $\min\left\{\mathrm{CoV}\left(B1^{+}\right)\right\}=\min\left\{\mathrm{std}\left(B1^{+}\right)/<B1^{+}>\right)\}$, where std denotes the SD. Finally, the combination of the magnitude and homogeneity was optimized using

(3) $$\min\left\{\mathrm{CoV}\left(B1^{+}\right)+\lambda \;<{\left(1/B1^{+}\right)}^{2}>\right\},$$

where λ is a regularization parameter.

To assess the prospect of deploying a universal shim in the neck, the convergence properties of universal neck RF shims were assessed using the data from the 10 volunteers. To test the results when calculating a universal RF shim for *N* (≤ 10) subjects, a candidate RF shim was calculated for the first *N* subjects, and its performance was evaluated using the multichannel B_1_^+^ data of all 10 subjects. The first *N* subjects were selected chronologically by acquisition date. For each comparison, tailored RF shims (where the shim is optimized for each subject separately) were included as an indication of the theoretical upper limit of the universal shim.

## 4 RESULTS

### 4.1 Wide dynamic range multichannel B_1_ ^+^ mapping

**FIGURE 3:**
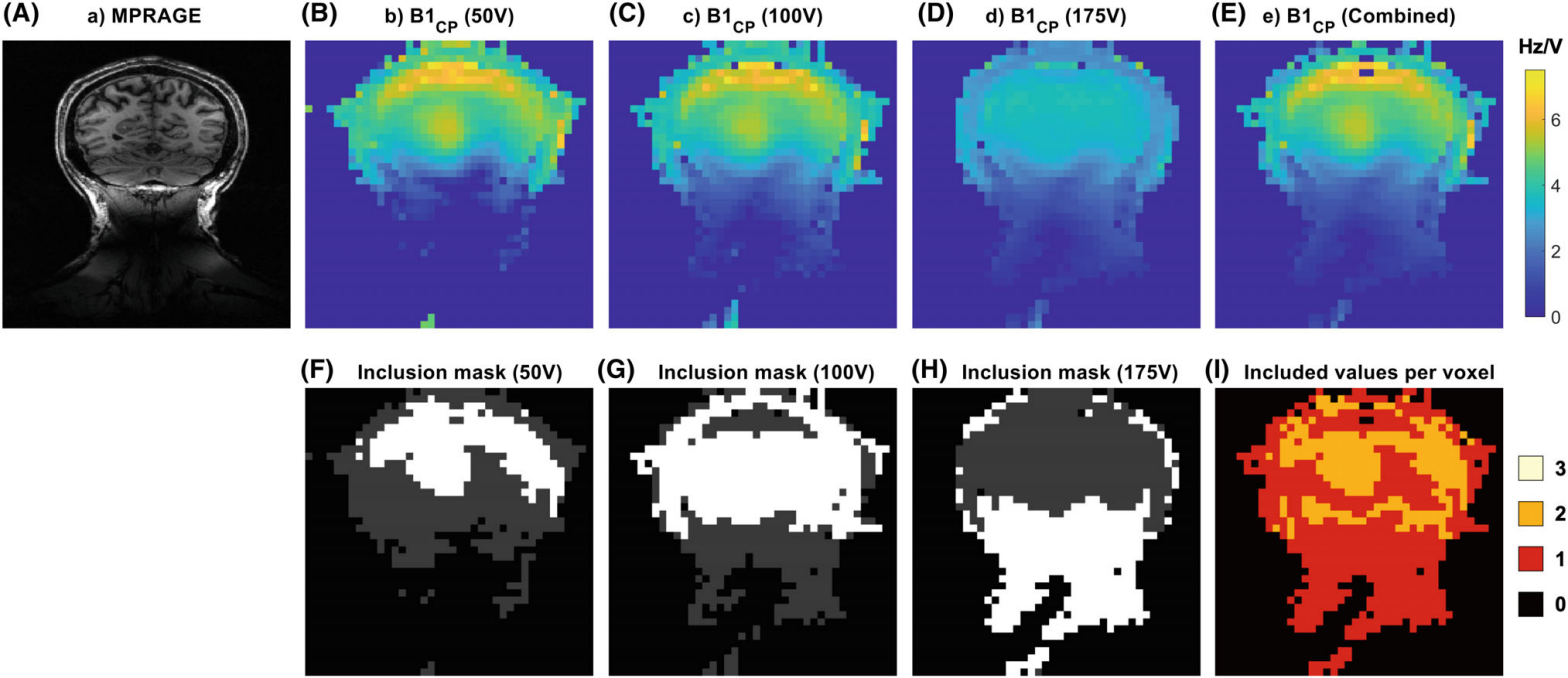
Absolute B_1_^+^ maps acquired at different voltages (B–D) are combined to obtain a single map with an increased combined dynamic range (E). Voxel values from individual scans were included or excluded based on the signal levels in the reference images and exclusion criteria to impose consistency in the acquired values relative to the other acquired data sets, resulting in inclusion masks for each data set (F–H). (I) The total number of included values for each voxel in the slice. CP, circularly polarized.

Figure 3 shows an example of combining absolute CP‐mode B_1_^+^ maps acquired using different transmit voltages when using the proposed exclusion criteria. Figure 3B–D shows the original CP‐mode B_1_^+^ maps acquired using transmit voltages of 50, 100, and 175 V per channel, respectively, and the resulting combined B_1_^+^ map is shown in Figure 3E. The masks of which voxel values were used to calculate the combined B_1_^+^ map (after application of the exclusion criteria) can be seen in Figure 3F–H, along with the number of values used to calculate the final combined B_1_^+^ map in Figure 3I.

**FIGURE 4:**
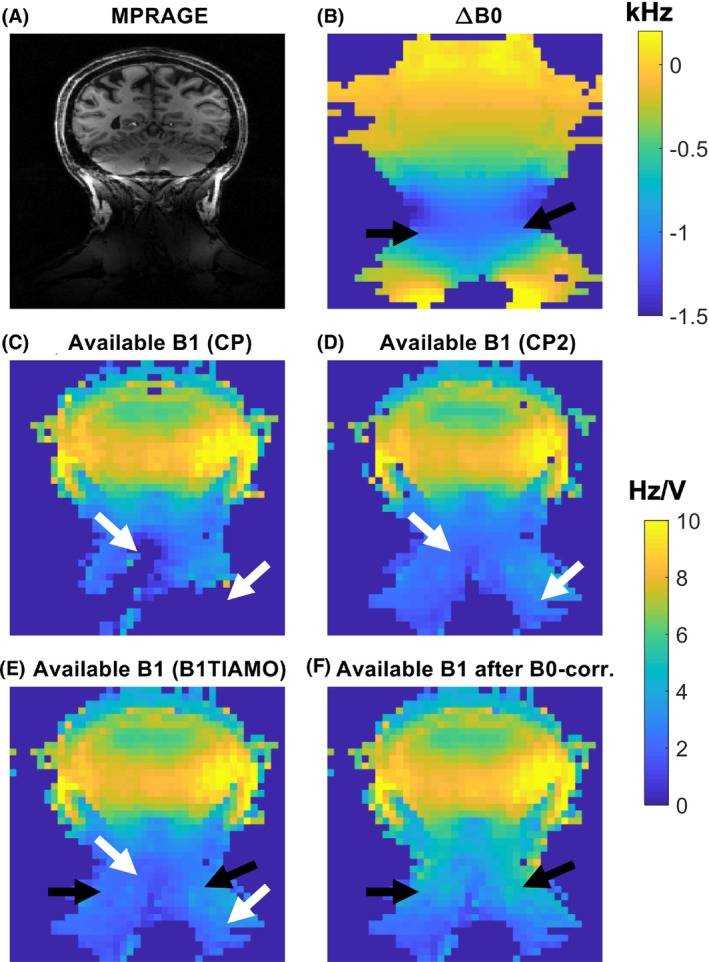
The use of B_1_^+^ time‐interleaved acquisition of modes (B1TIAMO) to mitigate signal loss in regions with low native B_1_^+^ in circularly polarized (CP) mode to obtain improved spatial coverage (C–E), and the consecutive correction for B_1_^+^ underestimation in the presence of high B_0_ inhomogeneity (F) based on measured B_0_ off‐resonance fields (B). White arrows indicate examples of improved spatial coverage due to B1TIAMO; black arrows indicate areas with substantial B_0_ offsets (up to −1.2 kHz), resulting in substantial B_1_^+^ underestimation if no B_0_ correction is applied. All B_1_^+^ data are shown in terms of the available B_1_^+^.

Figure 4 shows an example MPRAGE image along with B_1_^+^ maps from the first step in the B_1_^+^ mapping pipeline. B_0_ off‐resonance of up to −1.2 kHz in the neck results in a B_1_^+^ underestimation of up to 49% if not corrected, as seen in Figure 4B. Figure 4C–E demonstrates the utility of B1TIAMO to increase the spatial coverage in areas with low CP‐mode B_1_^+^. Figure 4E,F shows the changes caused by B_0_ correction of the absolute B_1_^+^ data based on the acquired B_0_ maps.

**FIGURE 5:**
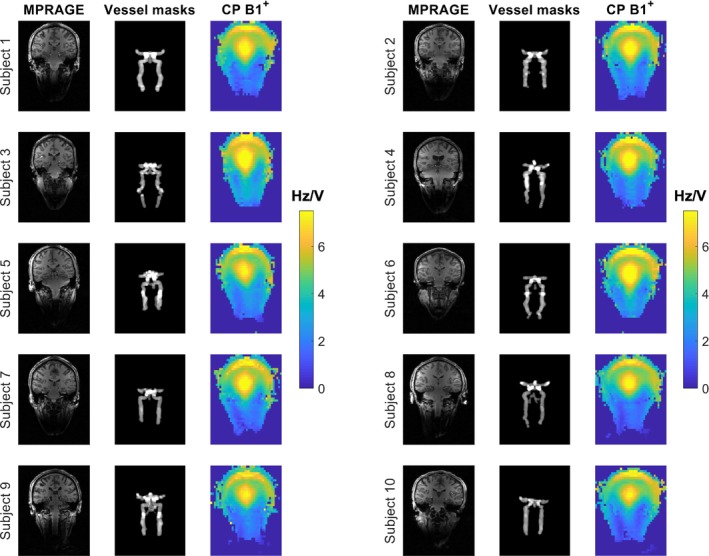
Central coronal slices of the 10‐subject database (doi: 10.5287/ora‐pvzkkddda) that was acquired using the proposed method. All data were acquired using the same FOV in coil coordinates. Left column, MPRAGE data (root sum of squares of circularly polarized [CP] mode and CP2 mode to improve structural visibility in the neck); middle column, coronal projections of the arterial vessel masks corresponding to the MPRAGE data; and right column, the CP‐mode B_1_^+^ map for each subject. The CP‐mode B_1_^+^ maps shown here are synthetic maps generated from the multichannel B_1_^+^ data.

An overview of the data acquired from the 10 healthy volunteers is shown in Figure 5. For each subject, a coronal slice of the MPRAGE data, a coronal projection of the hand‐drawn vessel masks, and the reconstructed CP‐mode B_1_^+^ map are shown. These data were acquired using a commonly used head pTx coil, so they could also be useful for other research centers for the calculation of universal pTx shims or pulses, or for simulation purposes. Therefore, the multichannel B_1_^+^ data and the B_0_ data are made openly available online (doi: 10.5287/ora‐pvzkkddda).

**FIGURE 6:**
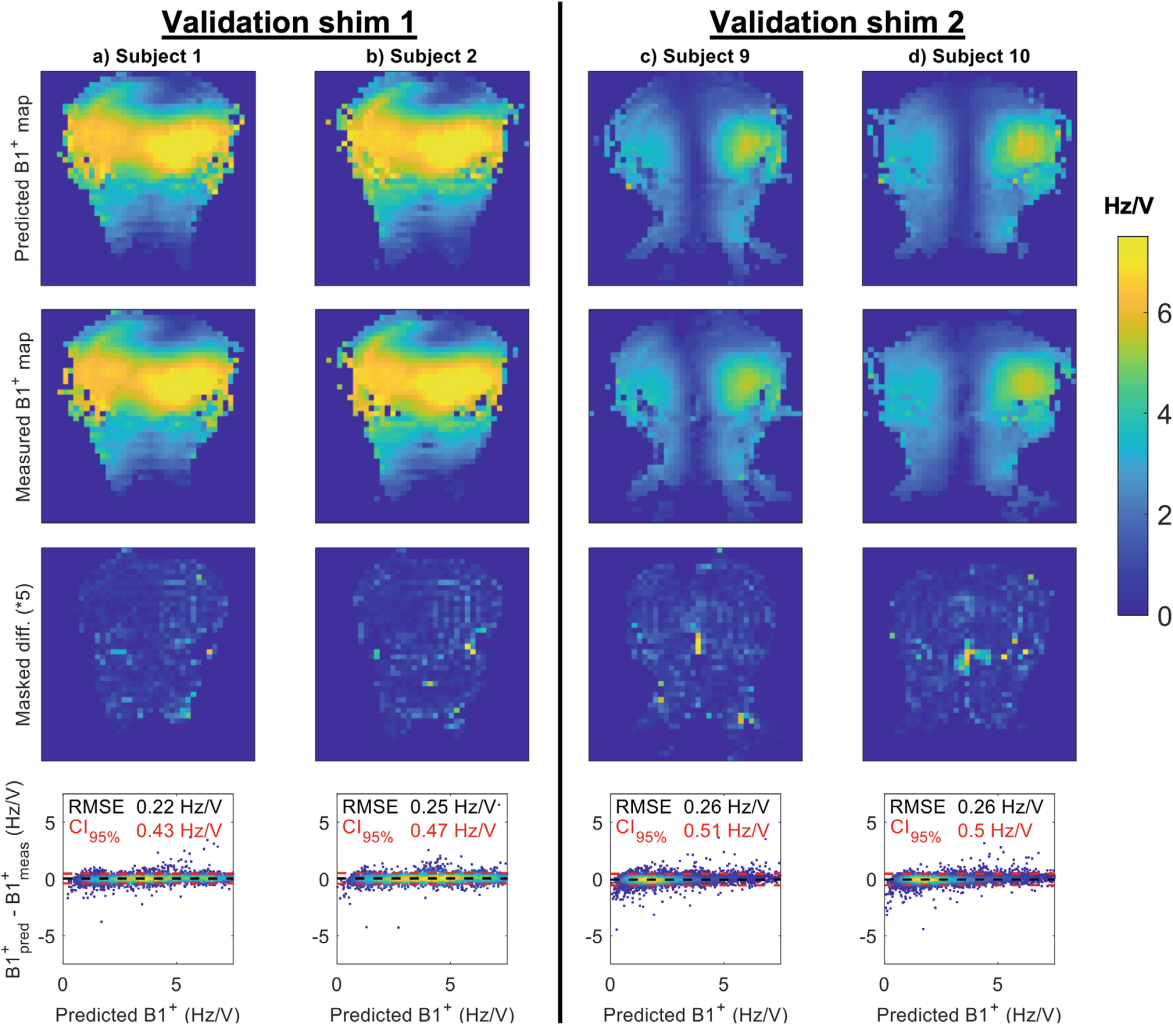
Evaluation of the agreement between predicted (first row, calculated from reconstructed multichannel B_1_^+^ maps) and measured (second row, acquired on the scanner using the same shim coefficients) B_1_^+^ magnitude maps for two arbitrary RF shims. The third and fourth rows show the absolute difference (using a 5‐fold boosted color scale) between the images in the first two rows, and the difference between the predicted (B_1_^+^_pred_) and measured (B_1_^+^_meas_) voxel‐wise values within the overlapping region. Dashed black lines indicate the mean errors, with dashed red lines indicating the means ±95%. Printed values indicate the RMS errors (RMSEs, black) and 95% confidence intervals (CI_95%_, red).

For Subjects 1, 2, 9, and 10, additional absolute B_1_^+^ maps were measured using two arbitrary RF shims. Figure 6 compares the measured (using the shim settings on the scanner) and predicted (combined multichannel B_1_^+^ maps using the corresponding shim coefficients) B_1_^+^ maps for those validation shims. Visually good agreement is found for all four comparisons, with a RMS error of 0.25 Hz/V across all comparisons and a median B_1_^+^ magnitude error of 3.8%.

### 4.2 Carotid artery RF shimming

The data from the 10 subjects shown in Figure 5 were used to study the potential B_1_^+^ benefits in the carotid arteries when using RF shims versus standard CP mode.

**FIGURE 7:**
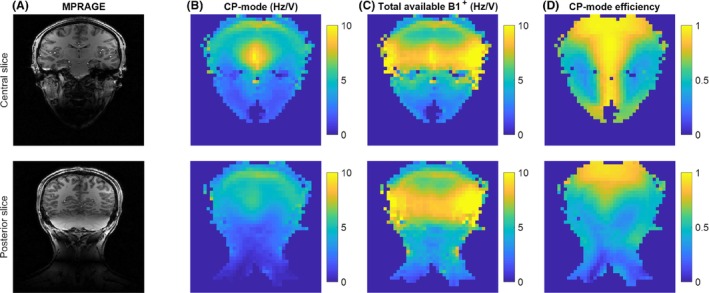
Two coronal slices from an example subject (Subject 1), showing a central slice (top row) and a more posterior slice (bottom row). Column B shows the B_1_^+^ map (Hz/V) in circularly polarized (CP) mode. Column C shows the maximum possible B_1_^+^ for each voxel (calculated as the sum of magnitude B_1_^+^ per channel). Column D shows the CP‐mode efficiency, calculated as the ratio of CP‐mode B_1_^+^ divided by total available B_1_^+^, indicating the loss of potential B_1_^+^ arising when using CP mode.

Figure 7 shows the CP‐mode absolute B_1_^+^, the total (theoretically) available B_1_^+^, and the resultant CP‐mode B_1_^+^ efficiency for two slices. Figure 7C shows that the theoretical upper limit of B_1_^+^ in the neck is (as expected) low compared with the central head region. In addition, Figure 7D shows that CP mode only uses 57% ± 5% of the theoretically available maximum B_1_^+^, resulting in an average B_1_^+^ magnitude in the neck for CP mode of 2.5 ± 1.0 Hz/V. This suggests that using pTx should be able to improve this very low B_1_^+^ penetration, albeit never realizing the theoretical maximum over a large region. A universal RF shim can be used to increase the average B_1_^+^ magnitude in the carotid arteries (as shown in Figure S1). However, the theoretical voxel‐by‐voxel upper limit indicated by the available maximum B_1_^+^ is unachievable using a standard RF shimming approach.

**FIGURE 8:**
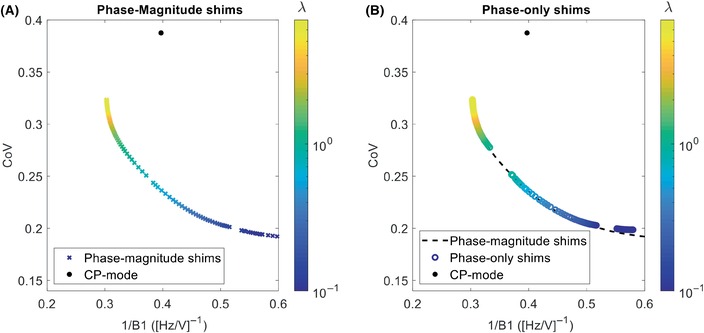
Plots investigating the trade‐off between the coefficient of variation (CoV) of B_1_^+^ within the vessel mask versus the achieved B_1_^+^ magnitude (expressed as 1/B_1_^+^). The desired regularization value, λ, that reduces CoV while retaining a strong B_1_^+^ is found at approximately *λ* = 1.7 (green region). (A) Universal shims that allow both phase and magnitude to change per channel. (B) Universal shims that allow only phase to change per channel. The dashed black line in (B) shows the data from (A) overlaid as a guide to the eye. The black dot indicates circularly polarized (CP) mode.

Maximizing only the B_1_^+^ magnitude without including a CoV constraint can result in B_1_^+^ inhomogeneity and large inferior–superior variation in the B_1_^+^ profile. Figure 8 shows the neck RF shim performance within the vessel mask when the CoV is included in the cost function using Eq. (3). Both magnitude‐and‐phase, and phase‐only B_1_^+^ shimming conditions were considered. Figure 8B shows that phase‐only shimming performs almost as well as magnitude‐and‐phase shimming, with nearly identical results (differences < 1%) when using a regularization parameter λ>1.5. Based on the L‐curve in Figure 8B, a regularization parameter λ of 1.7 is found to produce a reasonable trade‐off between B_1_^+^ efficiency and minimizing the CoV.

**FIGURE 9:**
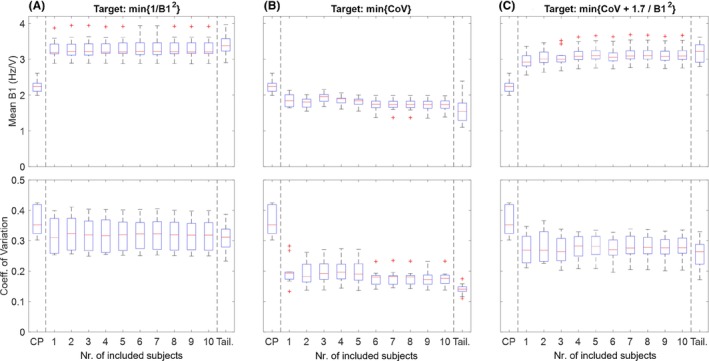
Plots showing the number of subjects needed to generate a universal neck shim (based on phase‐only RF shims). For each plot, the circularly polarized (CP) mode mean B_1_^+^ and coefficient of variation (CoV) are shown for reference, followed by the relevant metrics for universal neck shims generated from increasing numbers of subjects. The final column for each plot shows the result when per‐subject tailored shims are used (denoted as “Tail”). The different columns show the results for three different cost functions: (A) minimizing {1/B_1_^2^}; (B) minimizing {CoV}; and (C) minimizing the optimum combination of {1/B_1_^2^} and {CoV} with regularization value λ = 1.7.

Figure 9 evaluates the number of subjects required to generate a universal shim. Good results can already be achieved when universal shims are calculated based on a single subject, and (when using λ=1.7) no further improvement is observed when including more than 4 subjects. For all shim targets (B_1_^+^ magnitude optimized, CoV optimized, and optimized using Eq. [3]), universal shims perform only slightly worse than fully per‐subject tailored shims, and substantially better than CP mode. Additional leave‐one‐out comparisons, in which for each of the 10 subjects a universal phase‐only neck shim is calculated based on the other 9 subjects, provide an average increase in B_1_^+^ of 36% ± 14% relative to CP mode. The highest mean B_1_^+^ increase in the leave‐one‐out comparisons is 62% (Subject 2), and the lowest mean increase is 8% (Subject 6, which already had the highest average B_1_^+^ in CP mode).

**FIGURE 10:**
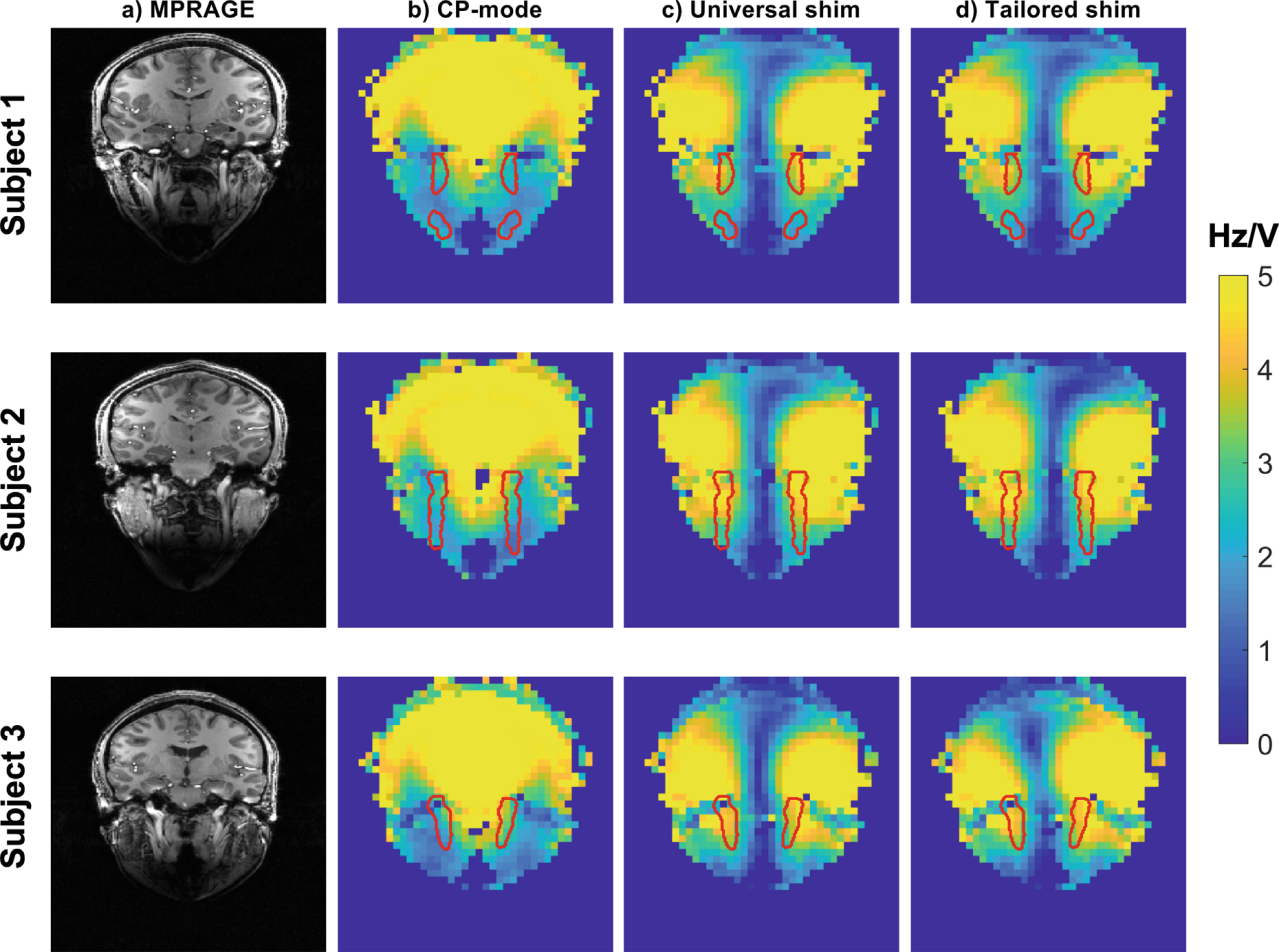
An example RF shim for the carotid arteries, shown for Subjects 1–3. Columns show an MPRAGE slice containing a superior segment of the internal carotid arteries (A); the circularly polarized (CP) mode B_1_^+^ in the same slice (B); the corresponding B_1_^+^ fields when using a phase‐only universal shim calculated using regularization *λ* = 1.7 (C); and the B_1_^+^ fields when using phase‐only tailored shims that are optimized for each individual subject, again calculated using regularization *λ* = 1.7 (D). The red outlines in (B) to (D) show in‐slice portions of the carotid masks used for shim calculation (at their original resolution corresponding to the MPRAGE data).

The final RF shim, calculated as a phase‐only universal shim based on all 10 subjects and using λ=1.7, is shown in Figure 10. Over the full carotid artery masks, this universal RF shim achieves an average increase in B_1_^+^ magnitude of 37% ± 16% relative to CP mode, while reducing the CoV by 26% ± 20%. When using tailored RF shims, the corresponding improvements relative to CP mode are a B_1_^+^ magnitude increase of 43% ± 20% with a 31% ± 20% reduction in CoV.

## 5 DISCUSSION

### 5.1 Wide dynamic range multichannel B_1_ ^+^ mapping

When combining B_1_^+^ maps acquired using CP mode and CP2 mode and with different transmit voltages using the proposed pipeline, a robust B_1_^+^ measurement can be obtained in the neck without compromising the B_1_^+^ accuracy in the head. Figure 5 shows that this increased coverage is consistently achieved, independent of subject size and position within the coil.

Figures 3 and 4 show that both the combination of multiple transmit voltages and B1TIAMO contribute to increasing the coverage of the final B_1_^+^ into the neck. The inclusion masks in Figure 3F–H show that data reconstructed from low‐voltage acquisitions are used primarily for the high‐B_1_^+^ areas in the center of the brain and close to the transmit elements, whereas high‐voltage data contribute primarily accurate information in the neck. These observations are consistent with the assumptions that motivated the use of multiple transmit voltages. Furthermore, they indicate that the exclusion criteria in Section 2, which do not make use of the spatial location of voxels, can accurately determine which transmit values to include at different spatial locations.

A single sandwiched satTFL acquisition using a transmit reference voltage of 60 V and using the same RF coil as this study has previously reported a dynamic range of a factor of 3 (ranging from 40° to 120°).^24^ Therefore, using transmit voltages of 50, 100, and 175 V per channel, the multivoltage approach used here can provide accurate results over a dynamic range of B_1_^+^ of a factor of 10.5, as the highest voltage of 175 V will enable B_1_^+^ regions that are as low as 13.7° at 60 V to be characterized accurately (60V/175V×40° at the lower end of the linear range of the method), and the lowest voltage of 50 V will enable B_1_^+^ regions that are as high as 144° at 60 V to be characterized accurately (60V/50V×120° at the upper end of the linear region of the method).

Despite the increased effective dynamic range when using multiple transmit voltages, even with a single shim (e.g., CP mode) no accurate B_1_^+^ information can be acquired in locations that have very low B_1_^+^ values due to destructive interference of the transmit fields. In such cases, Figure 4 confirms that including a CP2‐mode acquisition and B1TIAMO combination of CP‐mode and CP2‐mode data provides complementary information and therefore yields an improved spatial extent of the B_1_^+^ maps. However, further improvements might be possible using different calibration RF shims than CP mode and CP2 mode (such as designated neck shims) or additional RF shims in the B1TIAMO computation.

Although combining data acquired using different RF shims and transmit voltages provides B_1_^+^ information with a larger spatial extent, B_0_ off‐resonance effects can reduce the accuracy of the measured B_1_^+^ values. In this work, we used rectangular pulses that required an additional B_0_ correction step (Figure 4) to obtain accurate values in areas with high B_0_ off‐resonance (up to −1.2 kHz were observed). Alternatively, nonadiabatic broadband full‐passage hyperbolic secant pulses^27^ can be used for the presaturated TurboFLASH acquisitions^24^ to reduce the B_0_ dependence of the B_1_^+^ estimates.

When comparing predicted B_1_^+^ maps (reconstructed using the proposed pipeline) and measured B_1_^+^ maps (acquired directly on the scanner) for arbitrary RF shims (Figure 6), excellent agreement can be observed throughout the imaging volume, including the neck. The mean RMS error of 0.25 Hz/V indicates that some differences remain between the predicted and acquired maps. However, some of this remaining disagreement may be caused by inaccuracies in the measured B_1_^+^ maps rather than the predicted B_1_^+^ maps. For example, in the low B_1_^+^ regions in the middle of the head for Validation Shim 2, discontinuities that do not typically appear in B_1_^+^ maps are visible in the measured B_1_^+^ maps, whereas the predicted B_1_^+^ maps remain spatially smooth. This is also visible in the scatter plots in Figure 5, where some higher errors are observed for voxels with low predicted B_1_^+^ values.

The proposed multichannel B_1_^+^ mapping method requires a total of 10 separate acquisitions (six absolute B_1_^+^ maps, three sets of relative B_1_^+^ maps, and one B_0_ map) with a total scan time of 6:45 min for the reconstruction of a single set of multichannel B_1_^+^ maps. This additional scan time would be a limiting factor if acquiring subject‐specific field maps at the start of a clinical exam. However, this is not a limitation if using the method to acquire a B_1_^+^ database for the calculation of universal RF shims or pulses.

### 5.2 Carotid artery RF shimming

Figure 7 shows that the B_1_^+^ efficiency of CP mode is low in the neck (57% ± 5% along the carotid arteries), meaning that there is substantial opportunity for improvement using (universal) RF shims. Figure S1 confirms that a universal shim can substantially improve the carotid B_1_^+^, while showing a reduction in B_1_^+^ in the circle of Willis (which, depending on the application, may be an advantage or a disadvantage). However, there is also substantial B_1_^+^ variation along the vessel, suggesting that the shim performance can be improved by adding a B_1_^+^ homogeneity constraint.

The results in Figure 8 show that combining B_1_^+^ homogeneity optimization with B_1_^+^ magnitude optimization (using Eq. [3]) can improve the average homogeneity with a minimal reduction in B_1_^+^ magnitude. When using a regularization parameter, λ, of 1.7, the CoV is reduced by 25% while the average B_1_^+^ magnitude is reduced by only 5%. Figure 8B indicates that the results using phase‐only shims are nearly equal to those of magnitude‐and‐phase shims when using λ>1.5, indicating an inherent requirement for high B1^+^ use from all channels to achieve sufficient B_1_^+^ in the neck.

The universal shim convergence comparison in Figure 9 shows that a universal shim for the vessels in the neck can easily be found (even based on a single subject), with no further improvement in shim performance when including more than 4 subjects in the shim calculation. Furthermore, Figure 9 shows that universal RF shims perform almost as effectively as fully tailored per‐subject RF shims, while consistently outperforming CP mode in terms of both B_1_^+^ magnitude and CoV. The results in Figure 10 show that both tailored and universal shims result in similar B_1_^+^ profiles, explaining why the results in Figure 9 indicate that a shim calculated from a single subject can already provide reasonable results when used as a universal shim for all other subjects.

Figure 10 also shows that, using a universal RF shim and λ=1.7, the B_1_^+^ magnitude in the vessels in the neck can be increased by 37%, while reducing the coefficient of variation by 26%. This can be achieved using phase‐only RF shimming and does not require magnitude‐and‐phase RF shimming. These results are based on optimization of the B_1_^+^ over the entire region of the carotid arteries in the vessel masks in Figure 5. For some applications, in particular for ASL, excitation targets can consist of a smaller portion of these vessels, such as when only labeling in a certain plane or when using vessel‐selective ASL.^28^ In such cases, the optimization is less constrained, allowing for larger improvements in RF shim performance. For example, when only including the left internal carotid artery as a shim target, a phase‐only universal RF shim can simultaneously achieve a 43% increase in B_1_^+^ magnitude and a 42% decrease in CoV relative to CP mode (data not shown). When optimizing for a shim target consisting of the vessels within a single slice just below the carotid siphon (as used for pseudo‐continuous ASL), the increase in B_1_^+^ magnitude using a phase‐only universal RF shim improves to 62% (with a 55% reduction in CoV). This indicates a slightly larger improvement than the increase in B_1_^+^ magnitude in the same area when using dielectric pads instead of RF shimming, which were previously found to result in a 57% B_1_^+^ increase.^3^ Using this single‐slice shim, the minimum B_1_^+^ in the labeling plane across the 10 subjects increases from 1.7 to 2.9 Hz/V. When using 0.3‐ms pseudo‐continuous ASL labeling pulses of 15°,^5^ this corresponds to a reduction in the peak transmit voltage from 165 V to 96 V, thereby reducing the need to increase the TR^4^, ^5^ or use VERSE‐shimming^4^ to remain within SAR limits.^6^

It should be noted that the vessel masks used in this study were drawn based on the vasculature of healthy volunteers. Although the results presented here indicate consistently improved B_1_^+^ in the carotid arteries for subjects with typical (vascular) anatomy, both the B_1_^+^ fields and the locations of the vessels in the neck might be different for patients with nonstandard anatomies. Figure 10 shows a consistent increase in B_1_^+^ across both the left and the right side of the neck when using the proposed shim, with a decrease in B_1_^+^ in the center of the neck. Although morphological variations in the shape of the internal carotid arteries increases with age,^29^ a patient study into the variability of the medial location of the ICAs^30^ found that the ICAs of most (96.1%) patients are located within the lateral half on each side of the neck and would therefore be expected to achieve substantial B_1_^+^ improvements even for the universal RF shim. A total of 3.6% of the ICAs were found in the medial half of the lateral mass, where the B_1_^+^ in CP mode is similar to the B_1_^+^ using the proposed shim. The B_1_^+^ reduction in Figure 10 would only correspond to the location of the ICAs in the remaining 0.3% of patients, who had ICAs located medial to the lateral mass.

Furthermore, the results presented here are all based on simple RF shims that are constrained to the superposition patterns that can be achieved using the available transmit channels. Because the average B_1_^+^ efficiency of the proposed universal RF shim is 74% ± 3%, it is expected that further improvements can be achieved when using more advanced dynamic pTx pulses, where additional degrees of freedom are introduced by continuously changing the pTx coefficients in combination with the gradient waveforms and pulse amplitudes. Dynamic pTx can be used to further achieve improved B_1_^+^ homogeneity and/or localization. However, an advantage of using RF shims is that they can directly be implemented into existing sequences without requiring further pulse design or introducing sequence timing restrictions, thereby not increasing experimental complexity for existing sequences while still achieving substantially improved B_1_^+^ efficiency and homogeneity.

Finally, a combination of dielectric pads and pTx shimming could provide modest further improvements to the B_1_^+^ performance in the neck. Preliminary B_1_^+^ data (in a single volunteer), acquired both with and without dielectric pads, indicate that while RF shims provide better B_1_^+^ results than dielectric pads, a combination of dielectric pads and pTx shimming provides further improvements to the resulting B_1_^+^ in the neck. However, further work would be required to fully assess the performance of RF shims and the potential of using universal shims in combination with dielectric pads.

## 6 CONCLUSION

Combining B_1_^+^ data acquired using different voltages with CP‐mode and CP2‐mode RF shims allows the reconstruction of accurate multichannel head‐and‐neck B_1_^+^ maps for pTx head coils at 7 T. Using this, universal RF shims can be designed that increase the B_1_^+^ magnitude in the arteries in the neck by 37%, while also improving the homogeneity. This is possible using phase‐only universal RF shims, facilitating easy implementation in existing sequences at 7 T.

## CONFLICT OF INTEREST STATEMENT

Peter Jezzard is the Editor‐in‐Chief of *Magnetic Resonance in Medicine*. In line with COPE guidelines, he recused himself from all involvement in the review process of this paper, which was handled by an Associate Editor. He and the other authors have no access to the identity of the reviewers.

## Supporting information


**Figure S1.** Plots showing the average B_1_^+^ across all 10 subjects for circularly polarized (CP) mode (orange lines), for the total available B_1_^+^ (blue lines), and the B_1_^+^ achieved using a universal neck shim (yellow lines; calculated using the B_1_^+^ magnitude cost function 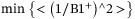). (A) The results averaged over the whole head volume (with the neck region indicated). (B) The data within the vessel masks only. Note that the B_1_^+^ superior to the neck mask is reduced for the universal shim relative to CP mode, whereas the B_1_^+^ within the neck mask is increased for the universal shim relative to CP mode.

## Data Availability

In support of *Magnetic Resonance in Medicine*'s reproducible research goal, both the *MATLAB* code for the multichannel B_1_^+^ reconstruction method (git.fmrib.ox.ac.uk/ndcn0873/b1_pipeline_reconstruction) and our 10‐subject multichannel B_1_^+^ database (doi: 10.5287/ora‐pvzkkddda) acquired using that method are openly available online. The online database also includes the corresponding B_0_ maps. In line with GDPR requirements, higher‐resolution structural MPRAGE data are available on request.
