# Supplementary material for: Head‐and‐neck multichannel B1 + mapping and RF shimming of the carotid arteries using a 7T parallel‐transmit head coil
Source: Magn Reson Med. 2023 Oct 5;91(1):190–204. doi: 10.1002/mrm.29845 (PMC10962593; doi:10.1002/mrm.29845)
Supplement: Supplementary file 1 — Figure S1. Plots showing the average B1 + across all 10 subjects for circularly polarized (CP) mode (orange lines), for the total available B1 + (blue lines), and the B1 + achieved using a universal neck shim (yellow lines; calculated using the B1 + magnitude cost function ). (A) The results averaged over the whole head volume (with the neck region indicated). (B) The data within the vessel masks only. Note that the B1 + superior to the neck mask is reduced for the universal shim relative to CP mode, whereas the B1 + within the neck mask is increased for the universal shim relative to CP mode. [file MRM-91-190-s001.docx]

**Supporting Information for “Head-and-neck multi-channel B1+ mapping and RF shimming of the carotid arteries using a 7T parallel transmit head coil”.**


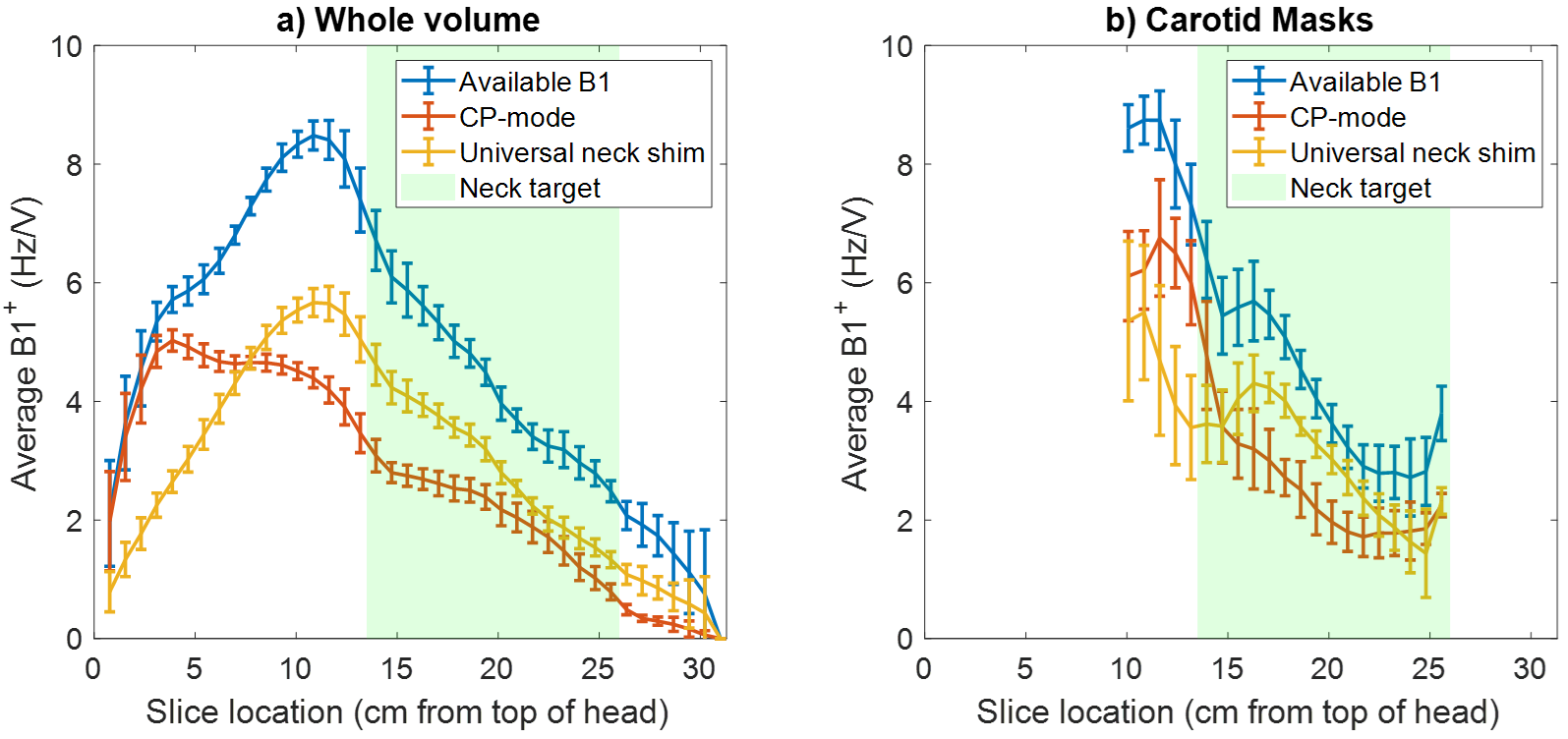


**Supporting Information Figure S1**: Plots showing the average B1^+^ across all 10 subjects for CP-mode (orange lines), for the total available B1^+^ (blue lines), and the B1^+^ achieved using a universal neck shim (yellow lines; calculated using the B1^+^ magnitude cost function $min\{<(1/B{1^{+})}^{2}>\}$). **(a)** shows the results averaged over the whole head volume (with the neck region indicated) and **(b)** shows the data within the vessel masks only. Note that the B1^+^ superior to the neck mask is reduced for the universal shim relative to CP-mode, whereas the B1^+^ within the neck mask is increased for the universal shim relative to CP-mode.
